# Commentary: Impact of COVID-19 pneumonia on pulmonary vascular volume

**DOI:** 10.3389/fmed.2023.1223819

**Published:** 2023-09-27

**Authors:** Vincent Jounieaux, Daniel O. Rodenstein, Yazine Mahjoub

**Affiliations:** ^1^Respiratory Department, Amiens University Medical Centre, Amiens, France; ^2^Respiratory Department, Cliniques Universitaires Saint-Luc, Université Catholique de Louvain, Brussels, Belgium; ^3^Cardiac Vascular Thoracic and Respiratory Intensive Care Unit, Department of Anaesthesia and Critical Care, Amiens University Medical Centre, Amiens, France

**Keywords:** COVID-19, SARS-CoV-2, intrapulmonary shunt, happy hypoxia, AVDS, pulmonary vasculature

## Introduction

We read with great interest the study by Fahrni et al. that was recently published in *Frontiers in Medicine* (1). In this paper, the authors performed automatic vascular volume extraction in ten chest CTs of COVID-19 patients with evidence of ground glass opacities. Vascular volume measurements included the whole vascular tree (i.e., pulmonary arteries, arterioles, capillaries, venules, and pulmonary veins, as well as bronchial vascularization to a lesser extent). Lung tissue affected by COVID-19 showed increased vascular volumes when compared to non-affected zones (respectively, 139 ± 82 mL vs. 46 ± 27 mL). Differences became significant when comparing the proportion of vascular volumes within diseased lung volume (8 ± 5%) to the proportion of vascular volumes within non-affected lung volumes (3 ± 2%, *p* = 0.026). The authors concluded that their results are consistent with other reports mentioning venous enlargement, increased lung perfusion in affected zones, and the supposed recruitment of pre-existing intrapulmonary arteriovenous shunts that could explain the discrepancies between the morphological disease severity on imaging and the clinical presentation of the patients.

## Commentary

Vascular disorders in COVID-19 infection are still investigated. Multiple vascular changes have been described including pulmonary embolism, vascular congestion or enlargement, perfusion changes and even pulmonary angiogenesis (2, 3). Since April 2020, we proposed the acronym “AVDS” (for Acute Vascular Distress Syndrome) to emphasize the target of SARS-CoV-2 (4). Indeed, COVID-19 infection appears more as a vascular rather than a pulmonary disease, characterized by an intrapulmonary shunt as observed in the hepatopulmonary syndrome (5). This intrapulmonary shunt explains all the characteristic features of COVID-19 infection: “happy” hypoxia (6), preserved pulmonary compliance and efficacy of prone position despite absence of pulmonary recruitment (7), relative inefficiency of pulmonary vasodilatators (inhaled nitric oxide) (8) and efficiency of pulmonary vasoconstrictors (Almitrine) (9).

## Discussion

Since its first description (4), the concept of AVDS is being increasingly confirmed (2, 3, 10) and the study of Fahrni et al. (1) is another support of this concept. To confirm our hypothesis that COVID-19 related vascular changes affect the lungs before appearance of alveolar condensations, it will be interesting to know if the team of G. Fahrni has proceeded to semi-automatic segmentation in control patients. If our AVDS concept is valid, one could expect that the proportion of vascular volume found in non-affected COVID-19 lung volumes (3 ± 2%) would appear increased when compared to the proportion of vascular volume in normal subjects. Moreover, it will also be relevant for Fahrni et al. to check the potential correlations between arterial blood gases (that were sampled in the study) and the proportions of vascular volume in COVID-19 patients. According to our findings, increased proportion of vascular volume with intrapulmonary shunt in such patients should be associated with lower PaO_2_ and lower PaCO_2_ (6), as in hepatopulmonary syndrome (5). Indeed, the intrapulmonary shunt characterizing AVDS seems related to lung vascular enlargements, aberrant angiogenesis with numerous arteriovenous anastomoses (10), and thus to the increased lung vascular volume. The considerable increase in pulmonary vascular volume (a 3.7 mean ratio of proportion of vascular volume between COVID-19 and non-COVID-19 affected areas) observed by the authors may support our data on right ventricular features in COVID-19 (11). We found a significant increase in right ventricular volume without any right ventricular dysfunction in non-intubated COVID-19 patients (without pulmonary embolism) (11). This suggests an adaptation of the right ventricle to an excess in volume load without increase in ventricular afterload as shown by Caravita et al. who found an increased cardiac output with decreased pulmonary vascular resistance (12). These features are similar to those found in hepatopulmonary syndrome (13). These points emphasize the work of Fahrni et al. and support our AVDS concept (14, 15). For all these reasons, the increased lung vascular bed volume observed by the authors (1) seems more the result of the SARS-CoV-2 infection rather than a pre-existing condition favoring the pathological manifestations of COVID-19.

## Author contributions

VJ, DR, and YM contributed to conception and design of the Commentary. VJ wrote the first draft of the manuscript. DR and YM completed sections of the manuscript. All authors contributed to manuscript revision, read, and approved the submitted version.
